# From their own perspective - constraints in the Polio Eradication Initiative: perceptions of health workers and managers in a district of Pakistan's Punjab province

**DOI:** 10.1186/1472-698X-10-22

**Published:** 2010-08-23

**Authors:** Muhammad Umair Mushtaq, Ubeera Shahid, Muhammad Ashraf Majrooh, Mushtaq Ahmad Shad, Arif Mahmood Siddiqui, Javed Akram

**Affiliations:** 1 Research Society, Allama Iqbal Medical College, Lahore, Pakistan; 2 Department of Health, Government of Punjab, Lahore, Pakistan

## Abstract

**Background:**

The success of the Global Polio Eradication Initiative was remarkable, but four countries - Afghanistan, Pakistan, India and Nigeria - never interrupted polio transmission. Pakistan reportedly achieved all milestones except interrupting virus transmission. This paper describes the perceptions of health workers and managers regarding constraints in the Polio Eradication Initiative (PEI) to ultimately provide evidence for designing future interventions.

**Methods:**

A qualitative cross-sectional study using focus group discussions and in-depth interviews was conducted in the Nankana Sahib District of Pakistan's Punjab province. Study subjects included staff at all levels in the PEI at district headquarters, in all 4 tehsils (sub-districts) and at 20 randomly selected primary health centers. In total, 4 FGD and 7 interview sessions were conducted and individual session summary notes were prepared and later synthesized, consolidated and subjected to conceptual analysis.

**Results:**

The main constraints identified in the study were the poor condition of the cold chain in all aspects, poor skills and a lack of authority in resource allocation and human resource management, limited advocacy and communication resources, a lack of skills and training among staff at all levels in the PEI/EPI in almost all aspects of the program, a deficiency of public health professionals, poor health services structure, administrative issues (including ineffective means of performance evaluation, bureaucratic and political influences, problems in vaccination areas and field programs, no birth records at health facilities, and poor linkage between different preventive programs), unreliable reporting and poor monitoring and supervision systems, limited use of local data for interventions, and unclear roles and responsibilities after decentralization.

**Conclusion:**

The study highlights various shortcomings and bottlenecks in the PEI, and the barriers identified should be considered in prioritizing future strategies.

## Background

Polio has crippled the human race for centuries, the first reports of the disease being made as early as 1350 [1]. In 1988, when polio was endemic in more than 125 countries affecting about 1000 children each day, the Global Polio Eradication Initiative (GPEI) was launched after a World Health Assembly resolution called for the eradication of polio by the year 2000 [2,3]. The success of the initiative was remarkable and nearly 5 million children were protected from being paralyzed by 2003 [4], however, four countries - Afghanistan, Pakistan, India and Nigeria - never interrupted transmission of the wild polio virus, and re-infection in twenty six countries in 2006-07 raised many concerns regarding the feasibility of polio eradication. By the end of 2007, polio incidence had decreased by 35% and polio transmission had stopped in all but 6 re-infected countries [5], yet polio eradication seems illusory as the endemic countries are missing polio eradication targets and 1648 cases were reported worldwide in 2008 [6].

Pakistan has a population of about 160 million and only 2% of GDP is spent on health [7,8]. Per 10,000 people, there are 8 physicians, mainly clustered in urban areas, and 4 community health workers. Public health care services are delivered by the provincial Departments of Health and Federal Government is concerned with planning and policy making. A district health system was introduced in 2001, and now districts are independent in administrative and financial matters regarding health care [9]. Pakistan adopted the Polio Eradication Initiative (PEI) within the Expanded Program on Immunization (EPI) in 1994 [10]. In Pakistan, PEI is managed at central level by the National EPI Cell, housed in the National Institute of Health at the Ministry of Health. It is concerned with planning, policy making, and the procurement of vaccines, the provision of technical guidance and mid-level management training, monitoring and evaluation, and coordination with international agencies. At provincial level, the PEI/EPI is managed by the Director of Health Services (EPI) in the office of the Director General of Health Services. He is concerned with the distribution of vaccines, supervision, monitoring and training. The Executive District Officer Health (or District Health Officer in some provinces) is the chief of the district health department and the pivotal person in managing the PEI/EPI with the assistance of a team of officials, explained later in the section on methods.

The number of polio cases in Pakistan decreased remarkably to 32 by 2007, including 19 WPV type 1 and 13 WPV type 3 cases with the virus found in only 18 of the 120 districts. But polio resurged in 2008, infecting 49 districts with 118 cases, including 81 WPV type 1 and 37 WPV type 3 cases - the highest number in the previous six years [4,6,10,11]. In 2009, 89 cases from 32 districts were reported and 15 high risk districts were identified, 9 having persistent transmission and 6 repeatedly infected districts, and district specific plans were developed [12]. Unprecedented support from the highest leadership was achieved, including the Prime Minister's Polio Action Plan in February 2009, which focuses on inter-sectoral coordination but it needs to be replicated at provincial and district levels and engage all sectors of civil administration and society [12]. Pakistan failed to interrupt polio virus transmission despite reportedly achieving the targets set in the GPEI strategic plan, including intensified mass immunization activities (national/sub-national immunization days, NIDs/SNIDs), mop-up campaigns within 4 weeks of every case, and certification standards for surveillance and laboratories. However, the Global Alliance for Vaccination and Immunization (GAVI) target of 90% national routine oral polio vaccine (OPV) coverage (current coverage of 83%) and 80% routine OPV coverage in every district by 2010 could not be reached [2,5,8,13]. The major factors contributing to the failure to interrupt virus transmission and the re-circulation of the virus were probably cross-border transmission from Afghanistan, poor commitment at the district level for polio eradication, sub-district coverage gaps, low routine coverage, operational weaknesses in the quality of services and large numbers of children missed during NIDs/SNIDs. Punjab has demonstrated the capacity to interrupt indigenous polio virus circulation and maintain the interruption for almost 2 years, but due to a convergence of factors, including insufficient EPI coverage, a reduction in the number of vaccination campaigns, and an influx of population from insecure areas having low immunity resulted in an environment favorable for the importation associated outbreak of polio in 2008 that continued in 2009 [12]. Ensuring a uniformly high routine OPV coverage and the high quality immunization campaigns, and sustaining the attainable achievements are the major challenges. The situation merits urgent attention.

As health programs mature, large-scale or national surveys become less useful. Instead, data from small-scale studies is required to evaluate different aspects of a program [14]. An evaluation of the PEI in one district of Pakistan's Punjab Province was conducted to establish a valid and reliable estimate for the effectiveness of polio eradication services and to provide scientific evidence for prioritizing future strategies. Both quantitative and qualitative methods were employed to provide an in-depth analysis of the factors contributing to failure to achieve polio eradication. The findings from the quantitative work that followed lot quality assurance sampling (LQAS) have been presented elsewhere and revealed low OPV coverage, both routine and during NIDs, and the poor quality of logistics management, monitoring systems and NIDs service delivery [15]. The main findings were [15]: five out of twenty lots were rejected for unacceptably low routine OPV coverage and the validity of coverage was questionable to the extent that all lots were rejected; among the 54% who were able to present immunization cards, only 74% had valid immunization; all of the 20 lots were rejected for poor compliance in logistics management and quality of monitoring systems; the mean compliance score and compliance percentage for logistics management were 5.4 ± 2.0 (scale 0-9) and 59.4%, while those for quality of monitoring systems were 3.3 ± 1.2 (scale 0-6) and 54.2%; 15 out of 20 lots were rejected for unacceptably low NID coverage by finger-mark; and all of the 20 lots were rejected for poor NID service delivery (mean compliance score = 11.7 ± 2.1 [scale 0-16], compliance percentage = 72.8%).

Very few studies have been conducted to reflect perspectives of managers and providers in immunization programs, focusing specifically on polio eradication [16-20]. The perceptions of those directly involved in the field can provide valuable information for designing future interventions. Qualitative assessment provides conceptual analysis, helping to understand the meaning of human social arrangements, program management services and decision making processes, and conveys to the policy makers the experiences of those executing the policies and those that are affected by the policies [21]. Therefore, the qualitative analysis was included in the evaluation with the aim of providing an in-depth performance analysis, and exploring the perceptions of health workers and managers regarding constraints in the PEI with reference to: 1) program resources and logistics, 2) technical aspects, 3) program operation, management and organization, and 4) monitoring, evaluation and feedback.

## Methods

### Study Area/Setting

The study was conducted in the Nankana Sahib District of Pakistan's Punjab Province. Punjab is the most populous province of Pakistan with 93 million inhabitants. There are 2,800 first level care facilities in the province, including about 300 rural health centers (RHCs) located in suburban areas and 2,500 basic health units (BHUs) located in rural areas, to provide primary health care services, including immunization [22,23]. Nankana Sahib District is situated in the central part of Punjab with an area of 2719 square kilometers. The District Department of Health provides curative, preventive and promotive services to 1.8 million inhabitants of the district, both rural and urban, with about 100 health facilities and 1800 health personnel. The district is divided into 4 tehsils (sub-districts), namely Nankana Sahib, Shahkot, Sangla Hill and Safdarabad, and 68 union councils. (Department of Health, District Nankana Sahib: Unpublished administrative data, 2009)

In Pakistan's Punjab province, the Executive District Officer Health (EDOH) is the head of the district health department and is assisted by the District Officer Health (DOH) for public health services. The PEI/EPI at district level is coordinated by the District EPI Focal Person, the District Polio Surveillance Coordinator (DSC) and the District Superintendent of Vaccination (DSV). At tehsil (sub-district) level, the Deputy District Officer of Health (DDOH) is responsible for public health services and the PEI/EPI is supervised by Assistant Superintendents of Vaccination (ASVs) and Inspectors of Vaccination (IVs). At town level, health care services are provided by a rural health centre (RHC) under the charge of a Senior Medical Officer (SMO) but no official is available there to over-see PEI/EPI activities. At union council level, the Medical Officer (MO) at basic health units (BHU) provides health care services and supervises vaccination. The Vaccinator is the pivotal person responsible for vaccination at union council level. In Nankana Sahib District, sanitation and communicable disease control (CDC) personnel, including the District Sanitary Inspector (DSI) and the Communicable Disease Control Officer (CDCO) at district level, the Tehsil Sanitary Inspector (TSI) and the CDC Inspector (CDCI) at tehsil (sub-district) level, the Sanitary Inspector (SI) and the CDC supervisor at the union council, are deputed for PEI/EPI activities along with their routine work. In addition, Lady Health Workers (LHWs) working under the National Program for Family Planning and Primary Health Care (NP for FP & PHC) are also deputed with vaccinators to improve vaccination coverage.

### Study Design and Sample

A qualitative cross-sectional study was conducted using Focus Group Discussions (FGDs) and In-Depth Interviews that have been extensively used in qualitative research in health care [21]. Qualitative design was followed to gain a more in-depth insight into the constraints in the PEI perceived by health workers and managers in a district. Qualitative methodology has been used commonly in investigating complex health care situations [24-29]. It helps to conceptualize the investigated area and its strength to assess processes and gain insight to the views of participants has been noted [26,30]. A complex intervention may operate differently in practice from the original intention and qualitative research can address how an intervention is used in practice [31].

#### FGDs for vaccinators (providers)

The target group was vaccinators. Two FGD sessions were organized. Each session involved 10 participants and a total of 20 vaccinators from 20 randomly selected primary health centers out of the 70 primary health centers in the district participated (RHCs at Syedwala, Warburton and Sangla Hills, and BHUs at Islamnagar, Youngsonabad, Chak 13 Randher, Chak 17 Karial, Bahawalkot, Nabi Pur Piran, Chak Hyderabad, Machhora, Marrar Chak 41, Marrar Chak 42, Marh Baluchan, Kot Rehmat Khan, Pakhariwal, Amer Kot, Qila Mir Zaman, Bahalike and Mandhiala).

#### FGDs for primary health center in-charges and tehsil (sub-district) managers

The target group included in-charges of the primary health centers and tehsil managers. Tehsil managers included ASV and TSI at Tehsil Nankana Sahib. The posts of DDOH, IV and CDCI in all the four tehsils and the posts of ASV and TSI in the other three tehsils of the district were lying vacant. Two FGD sessions were organized. Session 1 involved 12 participants and session 2 involved 10 participants, and a total of 2 tehsil PEI/EPI managers and 20 in-charges of the primary health centers (3 RHCs and 17 BHUs named above) participated in the sessions.

#### In-Depth Interviews of district managers

In-depth interviews were conducted with district managers of the PEI/EPI. The participants included: the EDOH, DOH, District EPI Focal Person, DSC, DSV, CDCO and DSI of the Nankana Sahib District.

### Data Collection

The guidelines for conducting the FGDs and interviews were developed and pre-tested and then revised based upon the feedback from the participants. The sessions were semi-structured but open discussion was encouraged. We probed participants on the following topics: program resources and logistics, technical aspects, program operation, management and organization, and monitoring, evaluation and feedback.

In total, 4 FGDs and 7 interviews were carried out. The FGD and interview sessions were conducted by a two member team that included a sociologist who acted as the facilitator, and a trained medical student who served to record the session and assist as required. The training and orientation session for the data collection team was held in Allama Iqbal Medical College, Lahore, Pakistan. The facilitator was responsible for recruitment, physical arrangements, conducting each session, analysis and reporting of the session. During each session, the facilitator made introductions, built rapport, encouraged discussion, controlled the rhythm, avoided being placed in the role of expert and summarized and concluded the session. The recorder kept a record of the contents of the discussion as well as emotional reactions and other important aspects. Individual session summary notes were prepared immediately after the completion of each FGD session and/or interview, and later synthesized, consolidated and subjected to content analysis. Data was kept confidential by assigning codes to the session notes, preventing unauthorized access and keeping the data secure and sealed after analysis by the Principal Investigator.

Verbal informed consent from the respondents was deemed sufficient due to the non-invasive nature of the study, but written permission from all the participants was taken prior to start of each FGD/interview session. Approvals for the study were granted by the Ethical Review Board of Allama Iqbal Medical College, Lahore, Pakistan and the Department of Health, Nankana Sahib District, Punjab, Pakistan.

### Data Analysis

Qualitative analysis of data is characterized by the development and manipulation of concepts [21]. Good practice for qualitative research, including theoretical sampling, validation and conceptual analysis was followed [32-35]. Data was analyzed by manual content analysis. The primary tasks were the inspection and coding and classification of data, and later subjecting it to conceptual analysis and development of typologies that convey the range of views, responses, or arrangements under study. Primary emphasis was paid to elucidating areas of thematic importance and exploring major constraints in failure to achieve polio eradication as perceived by the providers and managers of PEI/EPI services. The analysis was aimed at identifying technical, organizational and behavioral issues. After each session, the facilitator and recorder met to review and complete the notes taken during the session and to evaluate how the session went. A full report of the session was then prepared that reflected the session as completely as possible, using the participants' own words. Key statements, ideas and attitudes expressed on each topic of the session were listed. After the transcript of the session was prepared, the statements were coded, using the left margins. Any additional comments were written in the right margins. The questions and their responses were categorized according to the objectives to summarize the results. Draft reports of each session were developed in which responses were noted as per pre-established guidelines. A consolidated qualitative report was synthesized following the completion of all qualitative assessments.

## Results

The main constraints identified by the respondents were the poor condition of the cold chain in all aspects, poor skills and a lack of authority in resource allocation and human resource management, limited advocacy and communication resources, a lack of skills and training among staff at all levels in the PEI/EPI in almost all aspects of the program, a deficiency of public health professionals, poor health services structure, administrative issues (including ineffective means of performance evaluation, bureaucratic and political influences, problems in vaccination areas and field programs, no birth records at health facilities, and poor linkage between different preventive programs), unreliable reporting and poor monitoring and supervision systems, limited use of local data for interventions, and unclear roles and responsibilities after decentralization.

### Program resources and logistics

The respondents indicated the poor condition of cold-chain equipment especially at peripheral level.

*"An air-conditioned vehicle is not available in the district, and the situation is not much different in other parts of the country. There are problems in collecting vaccines from provincial stores in ice boxes especially in summer. There is a shortage of equipment in all aspects of the cold chain including freezers/ice-lined refrigerators, cold boxes, vaccine carriers, thermometers and even ice packs. Vaccines are distributed from district stores to tehsil (sub-district) and town (rural health center) level and then to a few basic health units. The vaccines are not stored in all union councils (basic health units) and many vaccinators collect the vaccines from the rural health centre. It results in cold chain failures due to the long distance travelled by the vaccinator to reach his area. At some places, the vaccination points for a union council are made at residential/private places instead of a health facility. This also affects the cold chain as temperature and correct placement of vaccines is often neglected there. The main reasons are equipment, electricity and security problems at health facilities." - Immunization managers and providers*.

The managers said that many shortcomings in resources and logistics could be addressed under the Punjab Millennium Development Goals (MDG) Program and the Punjab Health Sector Reforms Program (PHSRP) as the EPI is also a priority area in these programs. They indicated the lack of skills and authority in resource allocation and human resource management among managers. The participants highlighted limited resources for advocacy and communication campaigns at district level. They indicated that all the advocacy and communication campaigns including television and print media advertisements, brochures/posters and banners etc. are designed at central level and local communities' knowledge and attitudes are neglected in this regard. In addition, a large segment of population is missed when communication strategies are not tailored to local/district level circumstances.

### Technical aspects

The participants indicated that no public health training school exists for vaccinators before their induction into the health services. Lack of regular training and essential skills among PEI/EPI staff at all levels was also highlighted.

"There is training for mid-level managers but no training program exists for district PEI/EPI supervisory and field staff. There is virtually no pre- or in-service training of vaccination staff." - Immunization managers.

*"During time allocated for monthly training, the field staff has to submit their reports and administrative data so the basic purpose of training is often overlooked." - Immunization providers*.

The managers said that the field staff lacks very basic knowledge about polio eradication and OPV, have poor communication skills that often pose an obstacle to valid immunization coverage, and knowledge and skills to operate and maintain the cold chain is deficient. Recording and maintaining vaccine temperatures and recognizing the importance of doing so was cited as a problem in the field by the managers. The reporting and information system was considered unreliable by the managers. It was indicated by providers that managers do not stress the technical aspects. The stress is more on the administrative aspects and side issues during supervisory visits.

### Program operation, management and organization

The participants indicated the need to sanction more supervisory posts and lack of public health personnel was highlighted.

*"There are no sanctioned posts for the DSC and EPI Focal Person and officials are assigned these duties on a temporary basis in addition to their regular duties. The posts of DDOH are vacant in most of the Punjab. In Nankana Sahib District, the posts of DDOH in all 4 tehsils, the posts of CDCI and IV in all 4 tehsils, and the posts of ASV and TSI in 3 out of 4 tehsils are lying vacant, and officials are appointed on ad hoc basis. This is contributed to a severe lack of qualified public health personnel and poor health services structure in the country. At town (rural health centre) level, there is no official to oversee the PEI/EPI and IVs should be at each rural health centre to supervise town-level activities." - Immunization managers*.

Political and bureaucratic hurdles were identified as a constraint to effectively plan and administer services by the managers. The district managers highlighted the deficient training in planning and management of the staff responsible for micro-planning. The participants stated that performance in the PEI/EPI is not discussed at monthly facility meetings. Ineffective means of performance evaluation were also indicated by the respondents.

"The current system does not have an effective means of accountability and performance-based evaluation. The annual confidential reports (ACR) system is one of the institutional tools used to gauge performance, but unfortunately it is neither annual nor confidential, resulting in a lack of confidence in the mechanism." - Immunization managers.

*"Salary increments or transfer-postings are not performance-based, which affects the attitude of workers, as excellent or poor performance leads to no immediate gains or losses." - Immunization providers*.

The providers indicated the problems in the vaccination area and the vaccinator's field program.

*"The vaccinator usually visits a specific area once a month and there is no mechanism to cover missed children. Currently the vaccination area is based on union councils that are revenue divisions and in some cases the vaccinator has to cater for large populations spread over long distances. Some areas of a union council may be better covered by the nearby union council staff and vice versa." - Immunization providers*.

The participants said that immunization cards are not kept safely and often mislaid by parents resulting in a lack of valid information for monitoring. No law for birth registration with a health centre and weak linkage between various health sector programs was also identified as a problem by the participants.

### Monitoring, evaluation and feedback

Unreliability of reporting and poor monitoring systems was highlighted by the respondents.

*"Although the monitoring for national and sub-national immunization days (NIDs/SNIDs) is rigorous, routine services are neglected. A defined monitoring system from the federal to the union council level that includes a team of officials and a reporting system is in place, but the impact is not there because of a lack of coordination between central, provincial and district managers, a lack of effective supervision and motivation among staff, poor monitoring skills, unreliable reporting, poor quality of data and a lack of data use for action at all levels. The information collected is not properly used for evaluation of health facilities. The decisions to plan and prioritize immunization services are often not evidence-based." - Immunization managers*.

District managers also raised concerns about confused roles and responsibilities after the decentralization of health services in 2001. They stressed that making district health departments more resourceful in policy and planning, human resource management, resource allocation, monitoring and evaluation and financial matters, and developing clear guidelines for roles and responsibilities of officials at every level from the centre to the districts may help to improve services. Table 1 presents a summary of the constraints in the PEI, as identified by the health managers and workers.

**Table 1:** Perceptions of health workers and managers regarding constraints in the Polio Eradication Initiative

Component	Major issues
Program resources and logistics	*Poor condition of cold chain in all aspects
	*Poor skills and authority in resource allocation and human resource management
	*Limited advocacy and communication resources
Technical aspects	*Deficient skills and training among staff at all levels in almost all aspects of the program
Program operation, management and organization	*Deficiency of public health professionals and poor health services structure
	*Administrative issues including:
	*ineffective means of performance evaluation,
	*bureaucratic and political influences,
	*problems in vaccination area and field program,
	*lack of immunization cards,
	*no birth records at health facilities, and
	*poor linkage between different preventive programs
Monitoring, evaluation and feedback	*Unreliable reporting and poor monitoring systems
	*Limited use of local data for interventions
	*Unclear roles and responsibilities after decentralization

## Discussion

Respondents highlighted the poor condition of the cold-chain in all aspects. Previous studies have reported poor cold chain and logistics compliance [15,36-40]. Electricity and security problems at health facilities were revealed, consistent with previous studies [15,37]. An intact and efficient cold chain system is essential for quality immunization services, and problems identified in this study need to be addressed to assure that quality vaccines reach every child. A lack of resources and infrastructure is not an uncommon issue in developing countries, but poor skills and a lack of authority in resource allocation and management further aggravates the problem, as highlighted in this study. A lack of skills and authority in resource allocation and human resource management among managers was reported in a previous study in Georgia [16] and the need to train and authorize health managers in resource allocation and staffing decisions has been stressed [16,41,42].

Limited advocacy and communication resources at district level were indicated in the study. Currently, all advocacy and communication campaigns, including mass media advertisements and brochures/posters are made at central level in Pakistan. These resources could be decentralized to districts so that advocacy and communication plans could be tailored to local needs. Decisions to plan and prioritize immunization services have generally not been based on studies of the populations' knowledge, attitudes and practices regarding immunization. Had the data about local communities' knowledge and practices been generated and used, advocacy and communication interventions could have been more effective in reaching zero-dose children [43].

A lack of public health training schools and training programs for vaccination staff, deficient knowledge and skills among staff at all levels in the PEI/EPI in almost all aspects of the program, and deficient knowledge of staff in communication skills were highlighted in the study. Previous studies show that the knowledge gaps underlie low compliance with the vaccination schedules [44,45] and the quality of interaction between health workers and caregivers is decisive to ensure completion of the vaccination schedule [46-48]. Interventions aimed at fostering the communication skills of health workers and community outreach can significantly improve immunization coverage, as shown in previous international studies [49-51]. Capacity building of staff and both pre- and in-service training programs are essential to the success of the program. It was indicated that supervision is often focused on administrative rather than technical aspects, and this negatively affects providers' motivation and performance [41].

A deficiency of public health professionals and poor health services structure, administrative issues, including ineffective means of performance evaluation, bureaucratic and political influences, problems in vaccination areas and vaccinators' field programs, no birth records at health facilities, and poor linkage between different preventive programs were highlighted in the study. Poor work organization, and weak management structures and practices especially at peripheral level have been reported previously [16]. Without improving the public health services structure, the success of any preventive program seems elusory. There is a need to develop structures, processes and the skills of the existing work-force and authorize and train health managers in resource management [16]. Political and bureaucratic influences hinder effective planning and administration of services and usually there is undue interference in the transfer-postings of staff [18,52]. The need for other health programs to collaborate with immunization programs has been stressed previously. In particular, Lady Health Workers (LHWs) working under the National Program for Family Planning and Primary Health Care (NP for FP & PHC) can be deputed with vaccinators to improve immunization coverage [18,53].

The unavailability of immunization cards and deficient knowledge among parents to keep cards safely was also indicated. The lack of immunization cards has been indicated in previous studies [17,54-57]. A national vaccination card program could significantly increase coverage, especially its validity, and the cost of developing such a program would be offset by the savings resulting from not vaccinating children who are already up to date with their vaccinations [58].

The deficient knowledge of staff about monitoring, poor reporting compliance, and limited use of local data for interventions was highlighted. Previous studies have also reported problems in data quality and validity [15,26,59-63]. The lack of supervision and knowledge among responsible staff has been indicated previously [64]. In Pakistan, EPI records are not computerized and a previous study had shown the feasibility of a linked database system for immunization [65]. The data quality audits conducted in 27 countries, including Pakistan, revealed weaknesses in monitoring systems at all levels [61]. There is a need to see the quality of data and monitoring systems in a broader perspective, not only focusing on technicalities but also on support mechanisms [62,66,67]. Supportive supervision is critical in strengthening and enhancing performance at health facilities regarding polio eradication [26,68,69]. Previous experiences have shown significant improvements in data quality and monitoring systems as a result of data quality self-assessment and the use of data for action [70-72], and this strategy is also recommended in the Reaching Every District (RED) approach and the Global Framework for Immunization Monitoring and Surveillance (GFIMS) by the World Health Organization [72,73]. It was indicated that decentralization has resulted in unclear roles and responsibilities which are adversely affecting health system performance, accountability and staff motivation. Similar results have been reported in many previous studies [16,41,74-76].

The study was based on qualitative data that might be a limitation in itself. However, qualitative research can yield very useful information for action especially in disease specific programs [24-31]. The study did not follow a pre-existing conceptual framework but the findings provide a baseline picture of deficiencies. The paper describes the qualitative part of the in-depth PEI evaluation in a district and the consistency of the results with the quantitative part of the project provides additional evidence of validity [15]. Reporting bias may have confounded some of the responses, but previous studies report similar issues suggesting that results are externally valid [16,77]. Although the study was conducted in a rural district of Pakistan's Punjab province, the findings may be generalized to other rural areas in Pakistan in particular and South Asia in general, due to similar health system infrastructures, socio-cultural environments and topography. We recommend further studies to evaluate the findings of the study, for example, supportive supervision, immunization service crash programs involving some administrative measures within the current infrastructure, giving authority to districts in resource management etc., which can be further evaluated by cluster-control trials and pilot projects at district level.

## Conclusions and Recommendations

Perceptions of the field personnel who are actually involved in program execution render an invaluable insight into constraints, and guide policy makers to institute practical interventions. Shortcomings and bottlenecks in the PEI highlighted in the study are potentially important due to the failure to achieve polio eradication despite continuous efforts by the global community over the past two decades, and these should be considered in prioritizing future strategies. Based on the opinions and views of health managers and workers in the PEI/EPI in a district, the following recommendations may help to improve polio eradication efforts in the region:

• Cold chain equipment should be updated and maintained on a regular basis. Air-conditioned vehicles should be made available in all district headquarters. Electricity and security problems should be addressed and the vaccination point for a union council should be at the respective health centre. Reserve stocks of essential EPI equipment and supplies should be maintained at the district stores.

• District health managers should be authorized and trained in resource allocation and human resource management.

• Advocacy and communication resources should be decentralized and districts should design their advocacy, communication and social mobilization strategies according to the local circumstances.

• There should be training schools for vaccination staff, before their induction in each district. These schools could also be used for further training of PEI/EPI staff, which should be mandatory for their promotion. Focused training programs, held bi-annually, should be initiated for PEI/EPI staff at all levels. All relevant staff should be trained in face-to-face individual and group communication skills, and in techniques for using printed material with the clients.

• District health departments should be authorized to make resource management and staffing decisions. The provision of incentives to young medical graduates for joining public health services and establishing national public health services is also recommended.

• Administrative measures, including appraisal of excellent performance, redefining vaccination areas based on a 10 kilometers radius and/or 15,000 inhabitants rather than revenue circles, and making flexible vaccination field plans should be instituted. The posts of DSC and EPI Focal Person in a district and IVs in rural health centers should be sanctioned. A law for birth registration with health facilities should be constituted and a birth register should be maintained by Lady Health Visitors. This could significantly increase OPV zero-dose coverage. The linkage between preventive programs should be improved. In Nankana Sahib District a unique model of multi-purpose health workers is being followed where EPI, CDC and sanitation staff is working together for PEI/EPI activities, and this could be done in other districts of Punjab, ensuring that equal incentives are offered to all the staff working for the PEI/EPI. A national immunization card crash program should be initiated. There should be a mechanism in place to ensure accessibility of immunization cards to vaccinators and monitoring personnel, and immunization cards could be declared mandatory for admission to the schools.

• The staff at all levels in the PEI/EPI should be trained to improve monitoring, data quality assessment and the use of data for action. Staff should be encouraged to do realistic reporting, and records should be computerized and integrated with the district health management information system (DHMIS).

• District health departments should be made more resourceful in monitoring, evaluation, and financial matters and clear guidelines for the roles and responsibilities of officials at every level from the centre to the districts should be developed.

## Competing interests

The authors declare that they have no competing interests.

## Authors' contributions

All authors contributed significantly in all phases of the project in accordance with uniform requirements established by International Committee of Medical Journal Editors (ICMJE) and contributors who do not meet authorship criteria are listed in acknowledgements. All authors have read and approved the final version of manuscript.

## Pre-publication history

The pre-publication history for this paper can be accessed here:

http://www.biomedcentral.com/1472-698X/10/22/prepub
